# Evolution of Perceived Cohesion and Efficacy over the Season and their Relation to Success Expectations in Soccer Teams

**DOI:** 10.2478/v10078-012-0072-y

**Published:** 2012-10-23

**Authors:** Francisco Miguel Leo Marcos, Pedro Antonio Sánchez-Miguel, David Sánchez-Oliva, Diana Amado Alonso, Tomás García-Calvo

**Affiliations:** 1 Facultad de Ciencias del Deporte Universidad de Extremadura. Cáceres, España.

**Keywords:** cohesion, efficacy, success expectations, performance, soccer

## Abstract

The main goal of the study is to examine the evolution of players’ perception of cohesion and efficacy over the season and their relation with success expectations. The research sample comprised 146 male soccer players, aged between 15 and 19 years (M = 16.96, SD = .76). Diverse instruments were used to measure cohesion, perceived efficacy, and success expectations. The most noteworthy results show that players whose expectations do not match the team’s final performance will experience a negative evolution of their levels of perceived cohesion and efficacy, whereas players whose expectations at the start of the season match the team’s final performance in the classification will maintain their degree of perceived cohesion and efficacy. The main conclusion of the study is that coaches and sport psychologists should attempt to clarify the players’ basic goals of the season to create expectations that match what is expected from the team.

## Introduction

Most coaches and professionals linked to sports teams agree on the importance of both an adequate climate and good social relations, and the high perception of skill among team members in order to optimize group efficacy. Thus, many expert investigators in group dynamics have identified cohesion and efficacy as two of the most important properties within a sports team (Carron et al., 2002a; 2002b; Heuzé et al., 2006a; Leo et al., 2010b; Myers et al., 2004; Watson et al., 2001). Notwithstanding, there are few studies that have attempted to analyze the evolution of these psychological variables over the season, as well as what kind of variables could influence this process (Heuzé et al., 2006b; 2007).

Considering each of these concepts specifically, the term team cohesion is defined by Carron et al. (1998) as “a dynamic process that is reflected in part by the tendency of a group to stick together and remain united in the pursuit of its instrumental objectives and/or for the satisfaction of member affective needs”. As expressed in the definition, this concept is changing and variable over time and is affected by a series of environmental, personal, leadership, and team factors that promote the level of cohesion. If it is taken into account that one of team factors refers to the group’s desire for success, this indicates that the greater these desires and higher expectations of success, the higher will be the degree of cohesion shown by the players to achieve their goals.

In addition, within the conceptual model of Carron et al., cohesion consists of four dimensions based on two levels of distinction. The first level distinguishes individual attractions to the group and group integration; the second level distinguishes the task and social aspects of group involvement. Thus, four constructs are identified: Group integration-task (GI-T), Group integration-social (GI-S), Individual attractions to the group-task (ATG-T) and Individual attractions to the group-social (ATG-S). In our study we only used one of the levels, because the aim was to examine the evolution of task cohesion aspects, which reflects the degree to which group members work together to achieve common goals, and social cohesion, which reflects the degree to which team members empathize with each other and enjoy the companionship of the group (Carron and Ball, 1977; Carron et al., 1985; 2005) in different moments of the season. These dimensions change as a function of the diverse above-mentioned factors. Therefore, as seen in previous studies, success expectations can have a close relation with levels of player-perceived cohesion over the season (Leo et al., 2010a).

With regard to perceived efficacy, diverse studies have attempted to appraise this construct through players’ and coaches’ opinions (Alzate et al., 1997; Bandura, 1997; Chase et al., 1997; Lent and Lopez, 2002; Leo et al., 2010b). Thus, there are various types of efficacy related to the sport context (Beauchamp, 2007), among which are noteworthy coach-perceived efficacy and teammate-perceived efficacy, self-efficacy, and collective efficacy. Bandura (1997) first defined self-efficacy as an individual’s belief in his or her capacity to successfully organize and perform a specific task. Coach-perceived efficacy is the coach’s assessment of each player’s capacities and skills with regard to the good development of the play actions (Chase et al., 1997), whereas teammate-perceived efficacy is the players’ belief in the capacities of their teammates to adequately meet the demands of the sport (Lent and Lopez, 2002). Lastly, collective efficacy is defined as the group’s shared beliefs in their capacities to organize and perform the actions required to achieve certain goals (Bandura, 1997).

As with team cohesion, the perception of efficacy will evolve as a function of a series of antecedents. These sources of information may be past performance, success expectations, the group’s physiological state, leadership, the motivational climate, team cohesion, etc. (Bandura, 1997). Thus, as previously noted by Leo et al. (2010a), among the diverse antecedents of efficacy, success expectations, understood as the belief in the attainment of a series of goals or sports success, can play an important role in the perception of efficacy (Riggs and Knight, 1994).

The relevance to examine these aspects is due to the narrow relationship between cohesion and different types of efficacy (Heuzé et al., 2007; Kozub and McDonnell, 2000; Leo et al., 2010b; Paskevich et al., 1999; Ramzaninezhad et al., 2009; Spink, 1990) and these variables with respect to performance (Carron et al., 2002b; Heuzé et al., 2006a; Leo et al., 2010a; Myers et al., 2004; Watson et al., 2001). In accordance to this issue, several works have shown that players who perceived greater cohesion level and perceived efficacy achieve higher performance at the end of the league (Carron et al., 2002b; Heuzé et al., 2006a; Leo et al., 2010a; Myers et al., 2004; Watson et al., 2001). Therefore, it might be interesting to know how these variables progress during a season, and how success expectations, which have been shown as cohesion and efficacy antecedent might influence those variables (Beauchamp, 2007; Leo et al., 2010a). Moreover, it is important to note that this line of research has not been pursued much in the sport sphere, although some investigations have indirectly examined cohesion and efficacy in different moments over a season (Heuzé et al., 2006b; 2007; MacLean and Sullivan, 2003).

The most relevant studies about the evolution of cohesion and efficacy were carried out in diverse sports such as handball (Heuzé et al., 2006b; 2007), and basketball (Heuzé et al., 2006b). These works present a similar line of results: Heuzé et al., (2006b) and Heuzé et al. (2007) found that all the factors of cohesion decreased their levels as the season advanced and approached the end. Likewise, the levels of players’ perceived collective efficacy were higher at the start than at the end of the season (Heuzé et al., 2006b; 2007; MacLean and Sullivan, 2003). Our research at the same time that shows new information about evolution of those variables in football players, aims to examine whether success expectations perceived at the beginning of the season might influence the perception of cohesion and efficacy at the end of the league. Unlike most studies, this checking was developed through the composition of two groups regarding whether expectations was achieved or was not achieved and how dependent variables changes in both groups during the league.

Thus, the main goal of the study is to examine the evolution of players’ perception of cohesion and efficacy over the season and their relation with success expectations. Hence, as first hypothesis, we propose that the diverse factors of cohesion, perceived efficacy, and success expectations will undergo significant changes over the season. The second hypothesis states that success expectations will emerge as an antecedent in the evolution of cohesion and efficacy.

## Material and Methods

### Participants

The research sample comprised 265 male soccer players, aged between 15 and 19 years (*M* = 16.96, *SD* = 0.76). All the players who made up the sample belonged to the 15 federated teams that played in the XI group of the Sub18 National League, and each participant held a federative card with his personal and sports data. The final sample was formed by 146 players who completed the questionnaires at the start and at the end of the season, and the players who did not complete the two measurements, or who had completed them on different teams (due to a possible change during the season) were eliminated. The team coaches (*N* = 15), aged between 29 and 45 years, with at least 7 years experience, also participated in the study. The study received ethical approval from the University of Extremadura. All participants were treated according to American Psychological Association ethics guidelines regarding consent, confidentiality, and anonymity of responses.

### Measures

**Cohesion**. To assess cohesion we used the Spanish version of the Group Environment Questionnaire (GEQ: Carron et al., 1985), carried out by Iturbide et al. (2010). This instrument has 18 items grouped into four factors. Despite this issue, due that our aim was to analyze the evolution of social and task dimensions, we used the two global factors grouped items in *task cohesion* (9 items, i.e., “The team members unite their efforts to achieve the goals during the training sessions and the games”) and *social cohesion* (9 items, i.e., “The team members like to go out together”). The items are rated on a 5-point Likert-type scale. In this study, we analyzed internal consistency through Cronbach’s alpha coefficient, obtaining in the first measure values of .76 for task cohesion and .73 for social cohesion, and in the second measure values of .68 for task cohesion and .75 social cohesion respectively.

**Efficacy**. To measure self-efficacy, collective efficacy, and teammate- and coach-perceived individual efficacy, we elaborated a questionnaire based on the guidelines of Bandura (2006) for all the dimensions, which has been used in other studies (Leo et al., 2010a,b). This questionnaire measures *self-efficacy*, in which each player rates himself; *collective efficacy*, in which each player rates the team’s capacity; *teammate-perceived efficacy*, where each player rates all the other members’ efficacy; and *coach-perceived efficacy*, in which the coach rates each player. All the items were grouped into a single main factor that includes perceived efficacy in all stages of the game. The items are responded on a 5-point Likert-type scale in all cases. The measurement was carried out in diverse phases of the game, valuing technical and tactical aspects in the phase of attack and defense (i.e., “How do you rate yourself in the defense phase at a tactical level?”), the physical and psychological aspects (i.e., “How do you rate yourself in the mental and psychological aspects?”), and a last item of general rating of the player in the game (i.e., “In general, how do you rate yourself as a player?”). In the initial and final measurements, the scale yielded alpha values of ,73 and ,76 for collective efficacy, ,85 and ,80 for self-efficacy, ,80 and ,77 for teammate-perceived efficacy, and ,86 and ,87 for coach-perceived efficacy. All the factors obtained adequate internal consistency (Nunnally, 1978).

**Success expectations**. To measure success expectations, we asked each player at the beginning and near the end of the season about the position that he believed he would occupy in the classification at the end of the season, with a response range of 1 to 16.

**Classification**. To assess the final performance of the team, we used the final position of each team in the classification table at the end of the regular league.

**Expectations/Classification**. To assess the degree to which each team perceived expectations matched the final position achieved, we decided to use a quotient between each teams’ success expectations, using average player’s perceived matched at the start of the season and the final team position in the classification. As a result, the closer score was to 1, the more closely the expectations at the beginning matched the final team classification; and, contrariwise, the closer the score was to 0, the farther were the expectations from the final result achieved. But this indicator is greater than 1 if the final ranking of a team is better than success expectations that a player indicated at the beginning of the league.

### Procedure

The study was carried out using a correlational methodology, with a longitudinal design of evolutionary analysis which consisted of two measurements at two different points in time, analyzing a subpopulation or specific group across the time interval. The measurements were carried out at the start of the season (Measurement 1) and at the end of the season (Measurement 2) with approximately 20–22 weeks between them. Measurement 1 was carried out during the first third of the competition to ensure that the teams had competed together in several official games and within a three-week interval. Measurement 2 was carried out in the last third of the season, following the same guidelines used for Measurement 1.

With regard to the measurement procedure, first, the main investigator of the study contacted each one of the coaches of the teams that could participate in the investigation to request the inclusion of their teams in the study. They were informed about the goals and the procedure of the study that would be carried out if they agreed to participate in the investigation. In total, 15 out of the 16 teams that make up Group XI of the Sub18 League agreed to participate in the first and second administration of the questionnaires. The players were also informed about the goals of the investigation, emphasizing that their participation was voluntary and that their responses would be confidential.

To collect the data, we developed a protocol to ensure that data obtention would be similar in the two measurements and in all the participants involved in the investigation. The participants completed the questionnaires before the training session to avoid possible alterations due to the interactions that could arise during the sessions and that could affect the measurements. Measurements were conducted in the changing room, without the presence of the coach, individually, in an appropriate climate that allowed the players to concentrate without any kind of distractions. Completing the questionnaires took approximately 20 minutes; the main investigator was present at all times and emphasized that the players could ask for clarification of any doubts that might arise during the process.

### Analysis

Data was analyzed using the SPSS 18.0 software for diverse types of analyses to determine the relations among the variables. Firstly, various tests were conducted to determine the nature of the data. We used the K-S test for independent samples to verify the normality of the groups, the runs test for randomness, and Levene’s test for the homoscedasticity or equality of variances. As the data were shown to be parametric, we applied parametric tests in the data analysis. The techniques used for the study were factor analysis, reliability analysis, descriptive analysis, *t*-tests for related samples, and discriminant analysis.

## Results

### Descriptive statistics and comparison of means in two related samples

Table 1 shows the descriptive statistics of the variables of the study at both measurement times. In general, the means of the components of cohesion and of efficacy are high both at the start and at the end of the season. Only coach-perceived efficacy increased its scores at the end of the season. The participants expressed high success expectations at the start of the season but these scores decreased at Measurement 2.

To determine changes in the diverse variables over the season, we conducted a *t*-test for related samples (Table 1). In general, significant changes in the factor task cohesion were observed (*p* > .00), with higher scores at Measurement 1 (at the start of the season) than at Measurement 2 (at the end of the season.

Similarly, both the levels of collective efficacy and of teammate-perceived efficacy decreased as the season advanced (*p* > .05). In contrast, significant differences were observed in coach-perceived efficacy (*p* > .03), with higher levels at Measurement 2 than at Measurement 1.

Lastly, there were significant differences between the measurements of success expectations (*p* > .00). That is, the players had higher expectations at the start of the season, and their levels dropped at the end of the season. Therefore, at the beginning of the league, the players thought they would have a good season because they had high expectations, but at the end of the season, their expectations better matched what actually occurred.

### Analysis of differences

In view of the decrease in the levels of the variables analyzed, we decided to establish two groups as a function of the variable expectations/classification, differentiating the players whose expectations matched the performance (EMP), that is, their expectations at the start of the season in the classification table approached the final team classification, and the players whose expectations did not match the performance (ENP), that is, their expectations in the classification table distanced from the final team classification. Thus, by means of the median (*Md* = 0.72), the sample was divided into two groups: the set of data lower or equal to the median represents 50% of the players, and the data higher than the median represents the other 50% of the total sample.

In order to determine the changes in the variables over the season, we carried out a t-test for two related samples as a function of the variable expectations/classification. Table 2 shows the significant changes in the factors of social cohesion (*p* > .04) and task cohesion (*p* > .00), in both groups. In both cases, there are significant differences, with higher scores at Measurement 1 (at the start of the season) in comparison to Measurement 2 (at the end). However, there was no significant evolution in the cohesion variables of the players of the EMP group.

A similar effect was noted when analyzing the values of perceived efficacy, where both the levels of collective efficacy (*p* > .03) and of teammate-perceived efficacy (*p* > .04) decreased significantly between the start of the season and the end of the season in the ENP group. In contrast, no significant evolution was observed in the players of the EMP group in these variables, which remained stable.

### Discriminant analysis

To determine the set of variables that maximize the differentiation of the groups and help to predict matching expectations to performance, we used discriminant analysis. For this purpose, the variables that showed significant mean differences as a function of the expectation groups created (EMP and ENP) were entered in the discriminant analysis. The analysis showed that the statistics that analyze the significance of the discriminant function are adequate (Wilks’ λ =.839, *χ*^2^ = 24.875, *df* = 5, *p* < .01)

Table 3 shows the structure matrix created after the discriminant analysis. There are five factors that can discriminate the match between expectations and performance (those with a coefficient higher than .30). The factor with the highest discriminate capacity was social cohesion measured at the end of the season, with a structure coefficient of 0.64. Next was teammate-perceived efficacy, which was a negative predictor, that is, it discriminated the subjects whose expectations did not match the performance. Next came social cohesion measured at the start of the season and collective efficacy measured at the end of the season; both of them were positive discrimination, i.e., they discriminated the players whose expectations matched the performance. Lastly, was self-efficacy measured at the start of the season, which, as with teammate-perceived efficacy, was a negative predictor.

Of all the cases, 71.9% were correctly classified, which means that this function (and the variables that form it) is capable of predicting a very high percentage of cases.

## Discussion

The main goal of the study was to examine the evolution of players’ perception of cohesion and efficacy over the season and their relation with success expectations, and for this purpose, we examined the three hypotheses previously presented.

The first hypothesis proposed that the diverse factors of cohesion, perceived efficacy, and success expectations would undergo significant changes over the season. In this sense, in general, the scores of all the variables decreased over the season, that is, the levels of cohesion and perceived efficacy dropped as the end of the league drew near.

Significant changes were observed in the main factor of task cohesion, with higher scores at Measurement 1 than at Measurement 2. Taken a priori, these results do not seem logical as the first measurement was carried out at the start of the season when the players barely knew each other, and one would expect an increase of both variables as the season advanced. However, in most of the investigations (Heuzé et al., 2006b; 2007), as in this study, the first measurement was taken after starting the league, after three matches, when the levels of cohesion are very high because the players have more hopes at the start of the season and they unite their efforts to achieve greater heights. Therefore, the players have a greater predisposition to persevere to achieve a better performance. The levels of cohesion are lower at the end of the season because at this time, the players perceive the goals to be achieved either as closer or farther away, and the variables that unite the group to seek a better performance no longer seem so important. Similar results were found by other authors in sports such as handball (Heuzé et al., 2007) and basketball (Heuzé et al., 2006b), and they note that all the cohesion factors decrease their levels as the season advances and the end is nearer.

In the same vein, the levels of collective efficacy and of teammate-perceived efficacy decrease significantly from the start to the end of the season. The significant differences in success expectations between both measurements are also noteworthy, with expectations at the start of the season being higher than at the end. The same reasons used to justify the decrease in the levels of cohesion are valid to justify the fact that the scores of collective efficacy, teammate-perceived efficacy, and success expectations also decrease at the end of the season. The simple fact of performing the measurement at the start of the season increases the teams’ success expectations. Their desire to achieve the initially proposed goals also makes them perceive higher levels of efficacy in their teammates and in the group. At the end of the season, all the scores decrease, because the players’ perceptions of efficacy and expectations are more objective, that is, more realistic. Various authors like Heuzé et al. (2006b), Heuzé et al. (2007) and MacLean and Sullivan (2003) also observed a decrease in the levels of collective efficacy from the start to the end of the season.

In contrast, in coach-perceived efficacy, we observed significant differences, with higher levels at Measurement 2 (at the end of the season). This may be due to the fact that, at the start of the season, coaches are more cautious about their team’s efficacy and also, coaches always have a more objective and realistic view of their players’ efficacy.

Taking the above comments into account, the first hypothesis is confirmed, verifying that the levels of cohesion, perceived efficacy, and success expectations changed along the season, decreasing as the end of the league approached.

Due to the decrease in the levels of the variables of the study, we differentiated the players whose expectations were met from those whose expectations exceeded their final performance. Thus, we proposed the second hypothesis, which stated that success expectations would be a determinant of the evolution of cohesion and efficacy.

Firstly, we observed significant changes in the two main factors of cohesion, social and task cohesion, in the ENP group. In both cases, there were significant differences, with higher scores at Measurement 1 than at Measurement 2. This may be due to the fact that the goals proposed at the start of the season—such as staying in the category, being in the middle of the table, being at the top, etc.—may be unattainable or can no longer be attained, so the levels of cohesion drop. However, we did not observe any significant evolution of the cohesion variables in the players of the EMP group because, at this time of the season, they may still have been struggling to achieve their goals.

Therefore, when analyzing the values of perceived efficacy, both the levels of collective efficacy and of teammate-perceived efficacy decreased significantly from the start to the end of the season in the ENP players. In contrast, no significant evolution was observed in these variables in the EMP players because, as they were still struggling to attain the proposed goals, their perceptions of team efficacy and teammate efficacy were still high. Hence, we corroborated that the levels of cohesion and perceived efficacy decreased in the ENP players, whereas the EMP players maintained their degree of cohesion and perceived efficacy.

Lastly, discriminant analysis was used to determine which variables best discriminated between the EMP and ENP groups. After analyzing the results, we observe that both social cohesion and collective efficacy at Measurement 2 (end of the season) allow us to classify the players into the EMP and ENP groups. At the start of the season, social cohesion clearly discriminates the players whose expectations match the final performance and the players whose expectations do not match the final classification. In contrast, teammate-perceived efficacy and self-efficacy had a negative discrimination. Therefore, cohesion and collective efficacy, which are collective perceptions, discriminate which players will be classified as the EMP group, whereas individually perceived efficacy (self-efficacy, coach-perceived efficacy and teammate-perceived efficacy) discriminates the players who will be classified as the ENP group. In accordance to this, we can test the second hypothesis, which states that success expectations will emerge as a determinant in the evolution of cohesion and efficacy.

Thus, the main conclusion reached in this study is that the coaches should attempt to clarify the main goals of the season, both personal and collective, for the players (Leo et al., 2009; Senécal et al., 2008), in order to create expectations that match the team’s possibilities. If the players’ expectations exceed the team’s possibilities, then their levels of cohesion and perceived efficacy might decrease and therefore, their performance should also decrease. If the goals to be attained are clearly defined, each player’s expectations will match those of the group, and in this way, the levels of cohesion and efficacy should be more constant and the team’s final performance might be better.

In order to reaffirm these conclusions, it might be interesting to present some limitations of the study. In this regard, this work is developed with players at a learning stage and only two measurements throughout the season, so lot of information during the league that might influence these results might have been overlooked. Furthermore, only relationships at inter - individual level have been developed, so the association between variables at intraindividual and inter – teams level might give us valuable information regarding this research topic.

Therefore, it would be interesting to perform investigations with professional teams, because their orientation towards performance is much higher, and this might prevent the slight decrease in the number of players between the two measurements due to their absence from the training sessions. Moreover, three measurements could be performed throughout the season, which would provide another view of how the variables fluctuate over time. Finally, it might be important to perform works through multilevel analysis, because it would offer deeper relationships between those variables at intraindividual, inter – individual and overall, inter – teams level.

Another important prospective to follow in further investigations will be to develop studies under experimental methodology, to test whether working through success expectations might influence cohesion level and perception of efficacy by group, which might lead to an important finding in order to elaborate psychological issues at the highest sports level.

## Figures and Tables

**Table 1:** Means, standard deviations and analysis of differences at Measurements 1 and 2

	Measurement 1		Measurement 2		Differences Measurements 1 and 2

	*M*	*DT*	*M*	*DT*	
	
Social cohesion	4.00	.66	3.91	.67	.14
Task cohesion	3.76	.69	3.58	.68	.01
Collective efficacy	3.80	.56	3.71	.55	.03
Self-efficacy	3.91	.61	3.88	.51	.51
Teammate-perceived efficacy	3.75	.49	3.65	.43	.04
Coach-perceived efficacy	3.61	.68	3.74	.75	.03
Success Expectations	14.47	2.28	12.97	3.13	.00

**Table 2:** Means, standard deviations, and analysis of differences at Measurements 1 and 2 as a function of high and low group expectations/performance

	EMP					ENP				

	Measurement 1		Measurement 2		*p*	Measurement 1		Measurement 2		*p*
	
	*M*	*DT*	*M*	*DT*		*M*	*DT*	*M*	*DT*	
Social cohesion	4.08	.67	4.07	.62	.93	3.92	.64	3.75	.69	.04
Task cohesion	3.69	.68	3.62	.78	.43	3.98	.77	3.69	.71	.00
Collective efficacy	3.82	.58	3.77	.56	.38	3.78	.53	3.64	.54	.03
Self-efficacy	3.92	.56	3.81	.55	.12	3.96	.70	3.83	.62	.19
Teammate-perceived efficacy	3.65	.47	3.60	.50	.42	3.86	.48	3.69	.36	.04
Coach-perceived efficacy	3.72	.74	3.73	.78	.94	3.51	.57	3.66	.77	.11

EMP = Expectations match performance; ENP = Expectations do not match performance.

**Table 3:** Structure matrix of the discriminant analysis of the expectations/performance match

	Function 1
Final social cohesion	.64
Initial teammate-perceived efficacy	−.44
Initial social cohesion	.39
Final collective efficacy	.33
Initial self-efficacy	−.32
